# Is cardiac MRI stress imaging a cost-saving strategy for obese patients?

**DOI:** 10.1186/1532-429X-16-S1-O100

**Published:** 2014-01-16

**Authors:** J Ronald Mikolich, John Lisko, Brandon M Mikolich

**Affiliations:** 1 NEOMED, Hermitage, Pennsylvania, USA; 2 Sharon Regional Health System, Sharon, Pennsylvania, USA

## Background

False positive nuclear stress myocardial perfusion imaging(MPI) findings are known to occur in obese patients and women with large breasts due to attenuation artifact. A false positive MPI study may lead to unnecessary coronary arteriography(Cath). CMR stress perfusion imaging is not affected by obesity and has better spatial resolution than MPI, without radiation exposure. The CE-MARC study showed superiority of CMR stress compared to MPI, but did not include patients > 120 kg (mean BMI = 29.2 kg/m2) due to magnet bore size limitations. This study was designed to assess the cost benefit of CMR stress as an alternative to MPI for obese patients with chest pain.

## Methods

Concordance of MPI and Cath procedures were retrospectively assessed from data in an institutional cardiac imaging database. MPI procedures were classified as Positive (at least 1 region of ischemia or infarction) or Negative (no ischemia or infarction). Cath procedures were classified as Positive (at least 1 stenosis > 70%) or Negative (no lesion > 70%). Concordance of MPI and CMR stress was assessed from a cohort of patients with both a CMR stress and a MPI within a 6 month time frame. There was no patient weight limit for either procedure since a wide bore magnet was available for obese patients. Concordance for both patient cohorts were also analyzed by body mass index (BMI) < 25, 25-30, 30-35 and > 35 kg/m2.. From these data, using Medicare and private payor reimbursement data, analysis was carried out to compare the cost of the MPI versus cMRI stress perfusion strategies for obese patients with BMI > 35 kg/m2. Cost of Cath for false positive MPI and cMRI was included to assess the true cost of each strategy. Cost was computed assuming a patient population of 60% Medicare and 40% private insurance.

## Results

The concordance rate of MPI and Cath for patients with a BMI > 35 kg/m2 was only 26%, indicating that 74% of patients with a BMI > 35 kg/m2 had a MPI which resulted in a Cath which may have not been necessary. The concordance of MPI and cMRI stress was similarly low, at only 21%(14% true positive). 84% of the discordant MPI-CMR patients had a false positive MPI, yielding a Cath rate of 30%. Using an MPI strategy, the cost per 100 patients was $418,237(or $4,182 per patient). Using a cMRI stress strategy, the cost per 100 patients was $193,617(or $1,936 per patient).

## Conclusions

The majority of MPI procedures in obese patients result in false positive studies which lead to invasive coronary arteriography for clarification. The high incidence of false positivity is not observed with cMRI stress perfusion imaging. Although the per study cost of cMRI stress perfusion imaging is greater, the overall cost of the cMRI strategy is significantly less when compared to the MPI strategy for obese patients, due to the increased cost of Cath to evaluate false positive MPI studies. Cardiac MRI stress perfusion imaging is a cost-saving strategy for evaluation of obese patients with chest pain and suspected ischemia.

## Funding

None.

